# Structural brain changes in people with dementia receiving Cognitive Stimulation Therapy: Preliminary findings

**DOI:** 10.1016/j.ijchp.2025.100643

**Published:** 2025-10-24

**Authors:** Lucas M. Loureiro, Elodie Bertrand, Eelco van Duinkerken, Jerson Laks, Valeska Marinho, Iris Bomilcar, Renata Naylor, Gloria Wong, Aimee Spector, Daniel C. Mograbi

**Affiliations:** a Department of Psychology, Pontifícia Universidade Católica do Rio de Janeiro, Rio de Janeiro, Brazil; b Laboratoire Mémoire, Cerveau et Cognition (MC²Lab UR 7536), Institut de Psychologie, Université Paris Cité, Paris, France; c Post-Graduate Program in Neurology, Universidade Federal do Estado do Rio de Janeiro, Rio de Janeiro, Brazil; d Department of Medical Psychology, Amsterdam UMC, location Vrije Universiteit, Amsterdam, the Netherlands; e Institute of Psychiatry, Federal University of Rio de Janeiro, Rio de Janeiro, Brazil; f School of Psychology and Clinical Language Sciences, University of Reading, UK; g Department of Clinical, Health, and Educational Psychology, University College London, London, UK; h Institute of Psychiatry, Psychology & Neuroscience, King’s College London, London, UK

**Keywords:** Dementia, Cognitive stimulation, CST, structural MRI

## Abstract

**Background:**

There are growing numbers of people with dementia (PwD) globally, a syndrome with well documented impact on brain structure and connectivity that lead to cognitive impairment. Cognitive Stimulation Therapy (CST) is a non-pharmacological intervention for dementia with a strong evidence-base indicating positive effects on cognition and mood. There is, however, a lack of data on the effects of CST on brain structural and functional changes. This study aimed to analyze the impact of CST on brain cortical differences through the symmetrized percentage change (SPC) of surface area, thickness and volume measured through magnetic resonance imaging (MRI).

**Methods:**

Structural MRI was collected from 17 PwD who received CST and 11 treatment as usual (TAU) controls pre- and pos-intervention. The SPC of cortical structure was calculated for each participant. Freesurfer’s general linear model was used, and the resulting statistical maps were thresholded at *p* < .01. The maps were then corrected for multiple comparisons using Monte Carlo Z simulation with 10.000 iterations. The family wise error cluster-level correction for multiple comparisons threshold was set at *p* < .05 and p-values were further corrected using the Bonferroni method to correct for both hemispheres.

**Results:**

There were no statistically significant differences between CST and TAU groups regarding age, sex or years of education. Relative to TAU, the CST group exhibited a decrease in surface area in the left rostral middle frontal region (P_FWE_ = 0.019, Cohen’s δ of 1.764), in addition to an increase in thickness in the right supramarginal (P_FWE_ = 0.012, Cohen’s δ of -2.063) and postcentral regions (P_FWE_ = 0.038, Cohen’s δ of -2.11). No differences in volume SPC were found between groups. A statistically significant correlation was also found between cognition and the right supramarginal gyrus thickness (*p* = .017).

**Conclusion:**

This is the first study on the effects of CST on brain surface area, thickness, and volume. Our results indicate a change in cortical structure associated with CST. Reduced cortical thickness in the rostral middle frontal region may be related to the effects of CST on mood and stress. Increased thickness in the right supramarginal and postcentral regions may be due to increased abilities of phonological processing, verbal working memory, and somatosensory processing. More data, with larger samples, are needed to support these findings.

## Introduction

The impact of dementia on brain structure and connectivity is well documented, surpassing that of normal aging. A number of studies have shown more prominent atrophy in different brain regions and changes in brain connectivity (Filippi et al., 2017; Frau-Pascual et al., 2021). Additionally, the decline in cognitive function and consequent impairment in everyday functioning and autonomy are central clinical symptoms of dementia, along with behavioral changes (Jónsdóttir et al., 2023). It is estimated that the number of patients affected by dementia worldwide will rise significantly, driven by increases in life expectancy. Between 1990 and 2016, diagnosis increased by 117 %. In 2019, around 57.4 million people were affected by dementia globally, with this number expected to increase to 83.2 million in 2030 and to 152.8 million in 2050 (GBD 2019 Dementia Forecasting Collaborators, 2022; Livingston et al., 2020).

Cognitive Stimulation Therapy (CST) is the only psychosocial intervention recommended by the UK National Institute of Care and Excellence (NICE) guidelines (National Institute for Health and Care, 2018) to improve cognition, independence and well-being, due to its global evidence-base (Desai et al., 2024). It is also supported by the Alzheimer's Disease International (as seen in the (Alzheimer’s Disease International, 2022). CST is a brief psychosocial intervention consisting of 14 group sessions over seven weeks, used in over 29 countries and adapted to different languages and cultures (University College London [UCL], n.d.).

CST aims to mentally stimulate participants through multisensory stimulation and implicit learning, with reminiscence, fun, choice, developing relationships, and a focus on opinion rather than facts as central concepts (Morley & Cruz-Oliver, 2014; Rai et al., 2018). Recent meta-analyses have demonstrated positive impacts on cognition (Woods et al., 2023), and reductions in depression (Marinho et al., 2021; Saragih et al., 2022), indicating that this intervention can provide benefits in several outcomes. A study assessing the feasibility and efficacy of CST in a Brazilian sample also found improvements in functionality and mood in PWD who received CST compared to those who received treatment as usual (TAU) (Marinho et al., 2021).

Although improvements in cognition have been reported, the neural correlates of CST are under-examined. A few studies have explored the brain connectivity effects of CST through functional imaging (Liu et al., 2021; Qumars Behfar et al., 2023), but the structural differences mediated by the program are still unknown. Until now, brain morphology was assessed only as the total intracranial volume (Qumars Behfar et al., 2023), with the authors finding no differences pre- and post-CST intervention. A better understanding of the structural effects of CST may shed light into its mechanisms, indicating the way that the therapy works on improving cognition and help soften the impacts of dementia on patients.

Considering the above, the present study aimed to analyze structural brain differences between a group of PwD participating in the CST program compared to a treatment as usual (TAU) control group. This was done by examining the symmetrized percentage change (SPC) of the cortex in pre- and post-intervention magnetic resonance (MR) images.

## Methods

### Participants

Data reported here comes from a subsample that underwent MRI before and after the intervention in a feasibility trial (for full details on outcome measures and results of clinical and behavioral change, see Marinho et al. (2021)). In this study, an initial sample of 52 outpatient participants that attended the Center for Alzheimer's Disease of the Federal University of Rio de Janeiro (CDA-UFRJ) was recruited based on inclusion and exclusion criteria similar to previous CST studies (e.g. Spector et al., 2003). Inclusion criteria were: clinical diagnosis of dementia according to DSM-IV criteria (American Psychiatric Association, 1994); Mini-Mental State Examination (MMSE) scores between 10 and 24 (mild to moderate dementia)(Folstein et al., 1975). Exclusion criteria were: presence of any communication, sensorial or physical disability that could affect their participation in CST.

After participants and caregivers provided informed consent, individuals were consecutively allocated into groups (treatment as usual [TAU] or CST + TAU [CST]) using a random list generated by a computer program and after stratification for dementia severity (Clinical Dementia Rating, CDR, (A. L. Maia et al., 2006) scores).

The CDR is based on a semi structured interview of the subject and an informant, and the clinical judgment. The rating is calculated through a series of different cognitive and behavioral domains (memory, orientation, judgment and problem solving, community affairs, home and hobbies performance, and personal care). It can range from 0 to 3 points, with Grade 1 being an informative of mild cognitive impairment, and grade 2 indicating moderate cognitive impairment (Khan, 2016; Maia et al., 2006). The nature of the intervention prevented blinding participants to the group to which they were allocated. Nevertheless, outcome assessment and data analysis were conducted by researchers (EB, DM, and LL) blind to the intervention and without direct contact with the outpatient clinic.

For the neuroimaging data collection, additional exclusion criteria were: non-removable metal implants, implantable cardioverter defibrillators, tattoos, or claustrophobia.

### Treatment conditions

#### CST

The CST program has been described elsewhere (e.g. Spector et al., 2003), with the intervention in the current study being implemented according to the translated and adapted procedures described by Bertrand and colleagues (Bertrand et al., 2019). For the treatment group, the program was delivered by three researchers (VM, IB and RN), in groups with between 5 and 8 participants. The intervention was conducted over 7 weeks, twice a week, completing a total of 14 sessions. All sessions began with the group song, followed by a warmup exercise and a main activity based on that week’s theme (e.g. foods, childhood, numbers, orientation). Sessions were tailored to the groups’ abilities and to be as inclusive as possible. To facilitate attendance and reduce transportation costs and barriers, the two weekly sessions were run on the same day, separated by a short break. Each session took roughly 45 min.

In addition to CST, all participants allocated to this group also received the regular treatment from CDA-UFRJ (below).

#### TAU

TAU comprised regular visits every two/three months to a geriatric psychiatrist and cholinesterase inhibitors (AChEI) prescription. All patients received AChEI and no changes in prescription were allowed for both groups during the study.

##### Clinical and behavioral measures

Data on participants’ cognition, mood, quality of life and daily life activities were previously reported, with no differences between groups during baseline (Marinho et al., 2021). For the purposes of the current study, we explored whether changes in these outcomes were correlated with brain alterations. To achieve this, delta scores (post- minus pre-intervention scores) from the Alzheimer’s Disease Assessment Scale-cognitive subscale (ADAS-Cog) (Schultz et al., 2001), Cornell Scale for Depression in Dementia (CSDD) (Portugal et al., 2012), Alzheimer's Disease Cooperative Study – Activities of Daily Living Inventory (ADCS-ADL) (Cintra et al., 2017), and Quality of Life in Alzheimer's Disease (QoL-AD) (Novelli et al., 2010) were calculated. A brief description of the instruments can be seen below.

The ADAS-Cog (Schultz et al., 2001) assesses cognition and uses 11 tasks to evaluate cognitive domains, namely memory, language, command understanding, and praxis. Higher scores indicate lower performance.

The CSDD (Portugal et al., 2012) is a 19-item interview that evaluates current mood based on observed symptoms and signs thar occurred the week before the interview. The information is corroborated by an informant, and higher scores indicate higher depressive symptomatology.

For the assessment of the activities of daily living (ADL), the ADCS-ADL (Cintra et al., 2017) was used. This 24-item scale measure patient’s competence in basic and instrumental ADLs. Informants select the most appropriate option regarding the patient's level of ability, with higher scores indicating more preserved competence.

A quality of life (QoL) assessment was also included in Marinho et al. (2021). The QoL-AD (Novelli et al., 2010) addresses physical health, energy social relationships, and enjoyment of life in a 13-item questionnaire and a self- and informant-report version, both used in this study. Higher scores are suggestive of better QoL.

### Neuroimaging measures

An imaging protocol was performed in participants pre- and post-intervention on a 3T Siemens Trio Scanner. In this study, a 3D T1 Magnetization Prepared Rapid Acquisition Gradient Echo (T1-MPRAGE; repetition time: 2300 ms; echo time: 2,27 ms; flip angle: 8°; field of view: 250 mm; 1 mm isotropic voxels) was used. Images were corrected for scanner induced geometric distortion to improve the reliability of structural measure, and head movement was restricted by adding foam pads inside the head coil.

Each individual’s T1-MPRAGE was then processed using the longitudinal pipeline of FreeSurfer version 6.0 (http://surfer.nmr.mgh.harvard.edu). Firstly, an unbiased within-subject template was created using robust, inverse consistent registration (Reuter & Fischl, 2011; Reuter et al., 2010). By using the information of the within-subject template the reliability and statistical power of the cortical structural estimates over time are increased.

Several pre-processing steps, including skull stripping, transformation to Talairach space, atlas registration, spherical surface mapping and creation of cortical parcellations (Desikan et al., 2006), were performed for the scans of both time points, using the information of the within-subject template (Reuter et al., 2012). In the second step, the white matter was segmented, and surfaces were nudged into the direction of the gradient to find white and grey matter and the pial surface. The distance between the white matter and pial surface gives the vertex-wise cortical thickness, surface area, and volume. In this study, the SPC of cortical structure was calculated in order to assess longitudinal changes in surface area, cortical thickness, and volume. SPC is a normalized rate of change calculated as 100 * rate / average. Rate is defined as (structure at follow-up – structure at baseline) / (the time between baseline and follow-up), and average is defined as 0.5 * (structure at baseline + structure at follow-up). This equals a rate of change over time in thickness, surface area, or volume divided by the average of baseline and follow-up. This measure does not depend on total intracranial volume and is less dependent on baseline values than measures such as percentage change (Berry, 1989).

Given that the length of the intervention was 7 weeks, time between baseline and follow-up was calculated as time in days to be most accurate. Whichever time scale was used will not change the statistical results; it will only influence the mean SPC values. Lastly, to be able to conduct between-group comparisons of cortical structure, the resulting SPC maps for thickness, surface area, and volume were smoothed using a 10 mm full-width-half-maximum Gaussian kernel (as seen in other studies, e.g. Hu et al., 2024; Vilany et al., 2017) and then non-linearly warped to FreeSurfer’s fsaverage standard space template. For each participant, the analysis stream was checked manually for registration and segmentation errors and corrected if necessary. SPC values can either be positive or negative. A positive SPC is indicative of an increase in the cortical measure over time, whereas negative values indicate a decrease, therefore suggesting atrophy or loss. In this study, a higher SPC index suggest regions where the intervention program might have slowed or reversed cortical decline in comparison to the control group, therefore possibly demonstrating a neuroprotective effect of the intervention.

### Statistical analysis

Sociodemographic, clinical, and behavioral data were compared between groups with independent samples *t*-tests or chi-square tests according to the variable characteristics (continuous or binary respectively). Clinical and behavioral data were correlated to SPC values using Pearson correlation. For all analyses, α was set at 0.05, and the software IBM SPSS Statistics (version.29) was used.

For the analysis of between-group differences in cortical structure over the course of the study, the smoothed SPC maps in standard space were entered into the FreeSurfer’s general linear model, which is specifically designed to deal with non-parametric surface-based data. Here, we used the 2-sided GLM. The resulting vertex-wise statistical maps were thresholded at *p* < .01. Previous studies have shown that this threshold reliably controls for the risk of false-positive findings, without being overly conservative (Greve & Fischl, 2018). Then, the maps were corrected for multiple comparisons using Monte Carlo Z simulation with 10,000 iterations. The family wise error (FWE) cluster-level correction for multiple comparisons threshold was set at *p* < .05. The p-values were further corrected using the Bonferroni method to correct for both hemispheres.

## Ethics

The study was approved by a local research ethics committee (CAAE: 57,019,616.5.0000.5263) and all PwD and caregivers provided written informed consent to participate.

## Results

### Sample characteristics

Of the 52 patients, one participant from the CST group was excluded for having attended only two of the 14 sessions, and another participant dropped out of the intervention. From the control group, two participants were excluded due to absence of an available informant, and another participant dropped out of the study. Of the 47 participants left, 19 did not have neuroimaging data, either because of exclusion criteria or lack of availability to attend the imaging sessions. The final sample for the current study consisted of 28 participants (11 TAU + 17 CST).

Overall, the CDR ratings ranged between 1 (mild dementia) and 2 (moderate dementia). The TAU group consisted of 11 participants were allocated to the TAU group (5 females, 8 with a CDR 2, mean age of 75.0 ± 8.1 years and 7.4 ± 4.5 years of education), while 17 participants were allocated to the CST group (12 females, 7 with a CDR of 2, mean age of 78.2 ± 8.3 years and 9.5 ± 6.2 years of education). Both groups were similar in age (t [26] = −1.01, *p* = .321), years of education (t [26] = −1.07, *p* = .296), sex (χ2 [1] = 1.77, *p* = .184), CDR (χ2 [1] = 2.67, *p* = .102), and baseline CSDD scores (t [26] = −0.86, *p* = .400). Follow-up CSDD scores, however, were statistically different between groups (t [26] = 2.19, *p* = .038), with CST participants scoring lower and suggesting an effect of the intervention on the mood of participants (for full results see Marinho et al., 2021). Mean thickness (t [26] = −1.00, *p* = .324 for the left hemisphere, and t [26] = −1.29, *p* = .207 for the right hemisphere), total area (t [26] = 1.33, *p* = .195 for the left hemisphere, and t [26] = 1.96, *p* = .061 for the right hemisphere), and cortical volume (t [26] = −0.59, *p* = .559) in baseline were not statistically different between groups. For each subject, both cortical volume and area values were normalized for estimated total intracranial volume, in order to account for variations in head size. Sample characteristics can be seen in Table 1.

**Table 1:** Sociodemographic and clinical characteristics of participants.

	TAU (*n* = 11)	CST (*n* = 17)	*p* value
CDR (mild ( %)/ moderate ( %))	3 (27.3 %) / 8 (72.7 %)	10 (58.8 %) / 7 (41.2 %)	.102
Sex (Male ( %)/ Female ( %))	6 M (54.5 %) / 5 F (45.5 %)	5 M (29.4 %) / 12 F (70.6 %)	.184
Age (years)	75.0 ± 8.14	78.24 ± 8.33	.321
Years of education	7.36 ± 4.48	9.53 ± 6.25	.296
Days between scans	58.54 ± 3.53	59.12 ± 5.8	.772
CSDD (baseline)	1.91 ± 2.3	2.71 ± 2.47	.400
CSDD (follow-up)	3.09 ± 1.87	1.47 ± 1.94	.038
Mean left cortical thickness (mm)	2.29 ± 0.11	2.33 ± 0.12	.324
Mean right cortical thickness (mm)	2.28 ± 0.14	2.34 ± 0.13	.207
Cortical volume / eTIV ( %)	28.12 ± 1.64	28.55 ± 2.02	.559
Total left area / eTIV ( %)	5.62 ± 0.24	5.49 ± 0.24	.195
Total right area / eTIV ( %)	5.67 ± 0.24	5.48 ± 0.26	.061

*Note.* CDR = Clinical Dementia Rating; *M* = Male; *F* = Female; CSDD = Cornell Scale for Depression in Dementia; eTIV = Estimated Total Intracranial Volume.

### Neuroimaging data

We set out to evaluate SPC related to the CST intervention on cortical structure and therefore included surface area, thickness and volume, as they represent three distinct ways to study the cortex. The results presented are of 2-tailed GLM’s, FWE-corrected, and corrected for the assessment of 2 hemispheres. We did not correct for the three modalities included. The means are presented *As spc* in mm/day.

#### Surface area

**Fig. 1:**
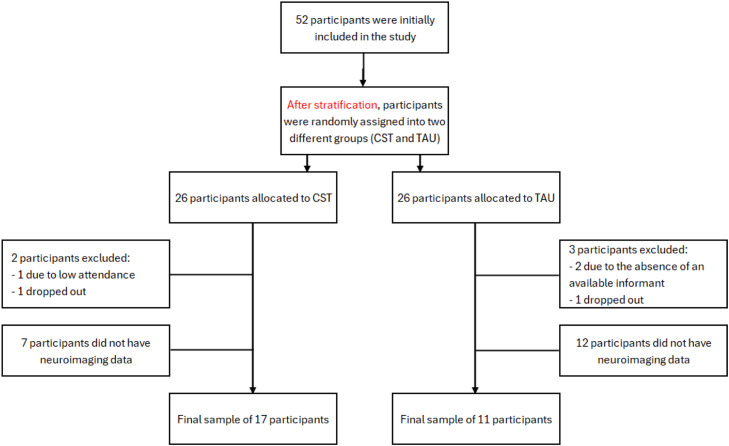
CONSORT diagram of our sample flow.

Regarding surface area, the CST group showed a cluster of decline compared to the TAU group. This cluster was located in the left rostral middle frontal region (Fig. 1), and was 123.30 mm^2^ in size, containing 145 vertices, with a maximum t-value of 4.126 (P_FWE_ = 0.019). The mean SPC in mm^2^/day for the TAU group was 0.118±0.10 % compared to −0.052±0.09 % in the CST group, resulting in a large effect size of Cohen’s δ of 1.764. There were no statistically significant clusters found in the right hemisphere. Fig. 1 shows the cluster with decline in surface area SPC, as well as the difference in surface area SPC between groups.

#### Cortical thickness

**Fig. 2:**
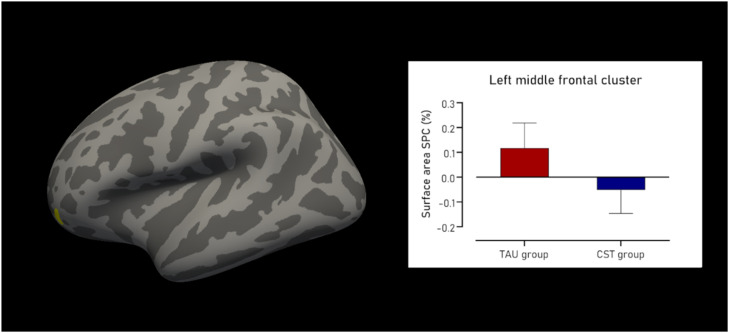
Cluster with decline in surface area and between group difference.

For cortical thickness, there were 2 clusters of increased indices in the right hemisphere for the CST group compared to the TAU group. The first cluster was located in the supramarginal and postcentral gyri (Fig. 2), was 174.01 mm^2^ in size, with 511 vertices, and a maximum t-value of −4.635 (P_FWE_ = 0.012). The mean SPC in mm/day for the TAU was −0.093±0.07 % and for the CST group 0.031±0.06 %, resulting in a large effect size of Cohen’s δ of −2.063.

The second cluster was entirely located in the lower part of the postcentral gyrus (Fig. 2), was 139.26 mm^2^ in size, with 372 vertices, and a maximum t-value of −4.894 (P_FWE_ = 0.038). The mean SPC in mm/day for the TAU group was −0.077±0.057 %, whereas for the CST group this was 0.026±0.043 %. This led to a large effect size of Cohen’s δ of −2.11.

**Fig. 3:**
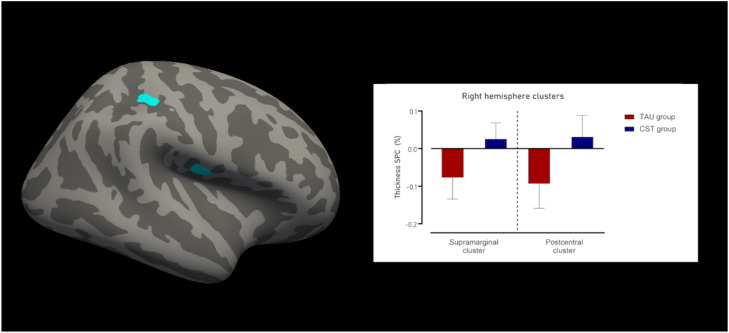
Clusters with changes in cortical thickness SPC and between group difference.

There were no statistically significant clusters found in the left hemisphere. Fig. 2 shows the clusters with changes in cortical thickness SPC, as well as the results from the thickness SPC analyses Fig. 3.

#### Cortical volume

No statistically significant clusters were found in either hemisphere for cortical volume.

### Correlations with clinical and behavioral data

Delta scores from the ADAS-Cog were significantly correlated with the right supramarginal gyrus thickness SPC values (r [26] = −0.45, *p* = .017). All correlation values can be seen in Table 2.

**Table 2:** Clinical/behavioral data and SPC correlations.

Variable	1	2	3	4	5	6	7
ADAS-Cog	—						
CSDD	−0.21	—					
ADCS-ADL	−0.56^⁎⁎^	−0.11	—				
QoL-AD	−0.24	−0.09	−0.23	—			
Rostral Middle Frontal Area SPC value	.19	.02	−0.26	.15	—		
Supramarginal gyrus thickness SPC value	−0.45*	−0.17	.34	.14	−0.49^⁎⁎^	—	
Postcentral gyrus thickness SPC value	−0.23	−0.26	.31	.16	−0.39*	.66^⁎⁎^	—

*Note.* ADAS-Cog = the Alzheimer’s Disease Assessment Scale-cognitive subscale; CSDD = Cornell Scale for Depression in Dementia; ADCS-ADL = Alzheimer's Disease Cooperative Study – Activities of Daily Living Inventory; QoL-AD = Quality of Life in Alzheimer's Disease.

*p < .05. **p < .01.

## Discussion

This study aimed to uncover the effects of CST on brain cortical structure. Therefore, we analyzed the MRI from participants that were randomly assigned to two different groups after stratification by dementia severity: one group received TAU, while the other additionally underwent CST over the course of 7 weeks. No statistical differences between groups were found for background variables, such as sociodemographic and clinical characteristics. Differences in SPC between groups were found in cortical thickness and surface area, but not in cortical volume.

The right supramarginal and postcentral regions, for which increases in thickness were found, are known to be involved in phonological processing and verbal working memory (Deschamps et al., 2014) and somatosensory processing (DiGuiseppi & Tadi, 2024). The effects of CST on language and verbal working memory (Hall et al., 2013), as well as an increase in connectivity in postcentral gyrus and other areas (Behfar et al., 2023) have already been reported, which may suggest a demand on both regions throughout the stimulation program. Regarding cortical volume SPC, no differences were found.

Interestingly, changes in cognitive performance, as measured by the ADAS-Cog, were correlated with increases in cortical thickness also in the right supramarginal gyrus, suggesting that greater structural preservation or enhancement was associated with better cognitive outcomes. The ADAS-Cog primarily assesses memory, exclusively in its verbal modality, as well as language functions (Schultz et al., 2001), both of which are closely linked to the supramarginal gyrus, a region implicated in phonological processing and verbal working memory (Deschamps et al., 2014). This convergence between cognitive domains targeted by the intervention and the functional role of the affected brain region reinforces the relevance of the structural changes observed.

These results are also in agreement with the previous literature on CST driven enhancement of syntax and verbal recall processing, potentially due to the language-based nature of the activities presented in the intervention (Hall et al., 2013). Current findings reinforce the notion that the language elements of CST may affect regions related to verbal cognitive processing. This highlights how the benefits in language ability provided by CST should be further explored. Most research into this field has been done with neuropsychological assessments, which provide fairly coarse assessments of language (Desai et al., 2024). Future studies may use tests specifically designed to explore in a more fine-grained manner linguistic ability (e.g. Montreal Protocol for the Evaluation of Communication Brief Battery [MEC B] (Casarin et al., 2019) or the Brief Montreal-Toulouse Language Assessment Battery [MTL] (Altmann et al., 2024)).

The decrease in surface area in the left rostral middle frontal region in the SPC of the intervention group compared to controls is a surprising result. This region is known to be important for social cognition (Amodio & Frith, 2006) and executive functioning (Mace et al., 2019). There is evidence from a family-based community sample that greater rostral middle frontal gyrus thickness is associated with perceived stress and sadness, whereas the opposite is true for positive affect (Michalski et al., 2017); given the mood benefits of exposure to CST in the Marinho et al. (2021) study, this may be a potential explanation for findings. Given the randomization procedure and the fact that the samples were matched, it is less likely this merely represents heterogeneity in the profile of neurodegeneration, but this possibility must be mentioned, especially considering the limited sample size.

There is a lack of data regarding brain surface area, thickness and volume in patients who underwent the CST program. Other studies have shown a cortical thinning related to dementia and subjective cognitive impairment in this region (Gerrits et al., 2016; Sattari et al., 2022; Youn et al., 2021), but the SPC in controls was found to be positive, whereas only the intervention group presented a negative SPC. As CST involves the use of executive function and social cognition of patients, more studies on brain morphology differences pre- and post-CST with more participants are needed to support this finding.

This study has several limitations. First, the small sample size limits the generalizing of findings, even though the effect sizes were large, and the statistical power to detect differences in all three metrics used in this study between the two groups. Therefore, these findings should be viewed as preliminary, and a larger sample size is necessary, which might be difficult due to the long-term nature of the CST program and the need to assess participants pre- and post-intervention. Despite this, the large effect size of these results can serve as a basis for novel studies. Second, the study lacked measurements of factors, such as comorbidities, diet and physical activity, and future studies should take the potential impact of these variables into account. Third, our sample consisted of people with different types of dementia, such as Alzheimer’s Dementia and Vascular Dementia. This heterogeneity could pose a problem when comparing different structural images. However, the randomization procedure was effective, so it is less likely that groups differed in relation to that. Lastly, another limitation is the lack of a follow-up neuroimaging assessment that might be helpful to establish the long-term effects of the CST intervention. Future studies should explore this, investigating the long-term effects, if any, of CST in brain structure and function.

The literature on structural and functional changes in the brain after CST is scarce, with this study being the first, to our knowledge, on the SPC of cortex measures. A finer understanding of these changes may reveal potential mechanisms of CST, leading to its development and refinement. By addressing the limitations of the current study in future research, a more comprehensive understanding of the structural brain changes associated with CST can be achieved, potentially informing more effectively the neural substrates of the intervention, further improving the quality of life for people living with dementia.

## Conclusion

In conclusion, this study demonstrates a change in brain surface area and cortical thickness in PwD that underwent a CST program, compared to controls. Moreover, results showed that greater structural preservation or enhancement in specific brain areas is associated with better cognitive outcomes. Further studies are needed to develop a better understanding of the effects of CST on brain structure.

## Declaration of competing interest

The authors declare that they have no known competing financial interests or personal relationships that could have appeared to influence the work reported in this paper.
